# Correction: Structural Changes and Lack of HCN1 Channels in the Binaural Auditory Brainstem of the Naked Mole-Rat (*Heterocephalus glaber*)

**DOI:** 10.1371/journal.pone.0149044

**Published:** 2016-02-04

**Authors:** The PLOS ONE Staff

## Notice of Republication

This article was republished on January 26, 2016, to correct errors in the figure order that were introduced while preparing the paper for production. The publisher apologizes for the errors. Please download this article again to view the correct version. The originally published, uncorrected article and the republished, corrected articles are provided here for reference.

## Supporting Information

S1 File Originally published, uncorrected article. (PDF)

S2 File Republished, corrected article. (PDF)
