# Supplementary material for: Correction: Structural Changes and Lack of HCN1 Channels in the Binaural Auditory Brainstem of the Naked Mole-Rat (Heterocephalus glaber)
Source: PLoS One. 2016 Feb 4;11(2):e0149044. doi: 10.1371/journal.pone.0149044 (PMC4742286; doi:10.1371/journal.pone.0149044)
Supplement: S1 File — (PDF) [file pone.0149044.s001.pdf]

PLOS | ONE

RESEARCH ARTICLE

Structural Changes and Lack of HCN1 Channels in the Binaural Auditory Brainstem of the Naked Mole-Rat (*Heterocephalus glaber*)

Nikodemus Gessele[1], Elisabet Garcia-Pino[1], Damir Omerbašić[2], Thomas J. Park[3], Ursula Koch[1]*

1 Neurophysiology, Institute of Biology, Freie Universität Berlin, Berlin, Germany, **2** Department of Neuroscience, Max-Delbrück Center for Molecular Medicine, Berlin, Germany, **3** Laboratory of Integrative Neuroscience, Department of Biological Sciences, University of Illinois at Chicago, Chicago, Illinois, United States of America

* ursula.koch@fu-berlin.de

CrossMark
click for updates

OPEN ACCESS

Citation: Gessele N, Garcia-Pino E, Omerbašić D, Park TJ, Koch U (2016) Structural Changes and Lack of HCN1 Channels in the Binaural Auditory Brainstem of the Naked Mole-Rat (*Heterocephalus glaber*). PLoS ONE 11(1): e0146428. doi:10.1371/journal.pone.0146428

Editor: Juan M Dominguez, The University of Texas at Austin, UNITED STATES

Received: August 12, 2015

Accepted: December 15, 2015

Published: January 13, 2016

Copyright: © 2016 Gessele et al. This is an open access article distributed under the terms of the Creative Commons Attribution License, which permits unrestricted use, distribution, and reproduction in any medium, provided the original author and source are credited.

Data Availability Statement: All relevant data are within the paper.

Funding: Funded by German Excellence Initiative and the Marie Curie Program of the European Commission - DRS postdoctoral fellowship (EGP). DFG - SPP1608, NeuroCure (UK).

Competing Interests: The authors have declared that no competing interests exist.

Abstract

Naked mole-rats (*Heterocephalus glaber*) live in large eu-social, underground colonies in narrow burrows and are exposed to a large repertoire of communication signals but negligible binaural sound localization cues, such as interaural time and intensity differences. We therefore asked whether monaural and binaural auditory brainstem nuclei in the naked mole-rat are differentially adjusted to this acoustic environment. Using antibody stainings against excitatory and inhibitory presynaptic structures, namely the vesicular glutamate transporter VGluT1 and the glycine transporter GlyT2 we identified all major auditory brainstem nuclei except the superior paraolivary nucleus in these animals. Naked mole-rats possess a well structured medial superior olive, with a similar synaptic arrangement to interaural-time-difference encoding animals. The neighboring lateral superior olive, which analyzes interaural intensity differences, is large and elongated, whereas the medial nucleus of the trapezoid body, which provides the contralateral inhibitory input to these binaural nuclei, is reduced in size. In contrast, the cochlear nucleus, the nuclei of the lateral lemniscus and the inferior colliculus are not considerably different when compared to other rodent species. Most interestingly, binaural auditory brainstem nuclei lack the membrane-bound hyperpolarization-activated channel HCN1, a voltage-gated ion channel that greatly contributes to the fast integration times in binaural nuclei of the superior olivary complex in other species. This suggests substantially lengthened membrane time constants and thus prolonged temporal integration of inputs in binaural auditory brainstem neurons and might be linked to the severely degenerated sound localization abilities in these animals.

Introduction

Naked mole-rats (*Heterocephalus glaber*) live in large eu-social colonies in narrow burrows underground and are exposed to low oxygen levels, almost complete darkness and an acoustically restricted environment during their entire life. In response to this distinct environment naked mole-rats have evolved physiological specializations of their peripheral and central nervous system leading to fundamental changes in the processing of sensory stimuli [1–5].

The extensive networks of narrow burrows have particular acoustic features. Low frequency sounds around 400 Hz propagate best and are at these frequencies even slightly amplified [6]. This is also reflected in the audiogram of the naked mole-rat, which is most sensitive between 500 and 1000 Hz and hearing of the naked mole-rat and is restricted to frequencies below 12 kHz [1]. A similar hearing range is found in other subterranean species such as the blind mole rat [7,8]. Acoustic signals propagated in burrows basically lack the typical binaural sound localization cues, such as interaural time and intensity differences. As an adaptation to this naked mole-rats display poor sound localization acuity and require long signal integration times to process binaural stimulus information [1,7]. However, naked and other mole rats are highly vocal and use a large repertoire of communication calls to exchange information in their colonies [9,10]. Moreover these communication signals are complex in terms of their temporal pattern and frequency fluctuations. Although naked mole-rats are exposed to such specialized acoustic surroundings very little is known whether their central auditory processing pathways show specific adaptations to this environment.

We therefore were interested whether binaural nuclei in the auditory brainstem, that analyze sound location, differ structurally and functionally in the naked mole-rat compared to other over-ground living rodents and whether monaural auditory brainstem nuclei, that analyze the temporal structure of sounds, e.g. communication sounds, are more similar in their features to other species. Since monaural and binaural sound analysis is largely dependent on the characteristics of excitatory and inhibitory inputs and the subsequent integrative properties of the neurons, we based our characterization of the auditory brainstem nuclei on the distribution of excitatory and inhibitory synaptic endings and the hyperpolarization-activated and nucleotide gated channel 1 (HCN1) channel. This voltage-gated ion channel greatly shapes the integrative properties of neurons and is highly enriched in binaural auditory brainstem neurons which integrate excitatory and inhibitory inputs on a very fast time scale [11,12]. Using a combination of antibody stainings against excitatory and inhibitory presynaptic markers we tried to unambiguously identify the major functional sub-regions of the auditory brainstem, based on the knowledge available from other mammalian species. Given the lack of localization cues and the large vocal repertoire of these animals one would expect degenerated auditory brainstem nuclei that process binaural localization cues, but normal to extended structures that analyze communication signals.

Material and Methods

Animals

Results for this study are from five 2–4 year old naked mole-rats (*Heterocephalus glaber*). Two animals came from our colony at the University of Illinois at Chicago and three animals were kindly provided from G.R. Lewin's colony at the Max-Delbrück Center for Molecular Medicine (Berlin, Germany). All Animals were housed in cages connected by tunnels, which were contained within a humidified incubator (40% humidity, 28–30°C), and heated cables ran under at least one cage per colony to allow for behavioral thermoregulation. Unlimited access to fresh fruits and vegetables was provided. For comparison of HCN1 staining four adult mice (C57/

B6) (~8 weeks) were used. These mice were housed in the animal department of the institute of Biology at 21±2°C, with a 12 hour light/dark cycle and food and water ad libitum. Animal protocols were approved by the German federal authorities (Landesamt für Gesundheit und Soziales, State of Berlin) or the Institutional Animal Care and Use Committee at the University of Illinois at Chicago.

Primary Antibody Characterization

Antibodies were used for both western blot and immunohistochemistry. Immunogen, host species, clone type, manufacturer's information, as well as dilution used for each antibody are listed in Table 1.

VGluT1 (vesicular glutamate transporter 1) was detected using a rabbit polyclonal antibody raised against Strep-Tag fusion protein of rat VGluT1. Its specificity was fully verified by the resulting negative immunolabelling in KO brain sections [13]. The staining pattern obtained with this antibody corresponds to what has been described in mouse sections including auditory brainstem [14–16]. This antibody is included in the Journal of Comparative Neurology primary antibody database (ID No. AB-887877).

HCN1 (Potassium/sodium hyperpolarization-activated cyclic nucleotide-gated channel 1) was located using a mouse monoclonal antibody raised against a fusion protein of rat HCN1. No cross-reactivity against HCN2 was found. Western blotting of HCN1 KO mouse brain membrane extracts revealed no bands in the immunoblots (manufacturer's data sheet). The staining pattern obtained with this antibody corresponds to what has previously been described in rat auditory brainstem nuclei (Koch et al., 2004). This antibody is also included in the Journal of Comparative Neurology primary antibody database (ID No. AB- 2115179).

GlyT2 (Glycine transporter 2) was examined using a guinea pig polyclonal antibody raised against synthetic peptide of rat GlyT2. Preabsorption of the antiserum with the immunogen peptide completely abolishes the immunostaining (manufacturer's data sheet). The staining pattern obtained with this antibody corresponds to what has been described for glycinergic neurons [18,19]. This antibody is also included in the Journal of Comparative Neurology primary antibody database (ID No. AB-90953).

MAP2 (microtubule-associated protein 2) was evaluated using a chicken polyclonal antibody raised against bovine MAP2. The antibody detects bands of the expected size of about 250kDa on western blots of mammalian brain tissue (data sheet and personal communication with Neuromics). This antibody stains neuronal cells in tissue, with the staining being limited to dendrites and perikarya. The antibody does not stain axons or any kind of glial cells, or hek293, HeLa, 3T3 cells. It has extensively been used as a neuronal marker in auditory brainstem neurons [19–21]. This antibody is also included in the Journal of Comparative Neurology primary antibody database (ID No. not available).

Additionally, we performed western blotting on brain samples collected from mouse and naked mole-rat in order to further test the specificity of the antibodies used in these uncommon research animals. Western blot analysis revealed a comparable band pattern of all the antibodies tested between both species (Fig 1), indicating that the antibodies also label the targeted proteins in the naked mole-rat brain.

Perfusion and tissue preparation. Animals were deeply anesthetized with an intraperitoneal injection of Ketavet (Pfizer; 100 mg/kg) and Rompun (Bayer Healthcare; 4 mg/kg), then perfused transcardially with 0.1 M phosphate-buffered saline (PBS; pH 7.4; 10 min) followed by 4% paraformaldehyde in 0.1 M PBS (30 min). After perfusion, brains were immediately dissected out of the skulls, post-fixed for an additional hour and washed in 0.1 M PBS.

Table 1. Primary antibodies used for the study.

Antibody	Immunogen	Manufacturer	Code	Host clonality	Dilution
VGluT1	Strep-Tag fusion protein containing amino acid residues 456–560 of rat VGlut1	Synaptic Systems	135 303	Rabbit, polyclonal	1:1000
GlyT2	synthetic peptide from the carboxy-terminus of rat GlyT2	Merck Millipore	AB1773	Guinea pig, polyclonal	1:1000
HCN1	fusion protein containing amino acids 778–910 (cytoplasmic C-terminus) of rat HCN1	NeuroMab	75–110	Mouse, monoclonal	1:1000
MAP2	bovine MAP2	Neuromics	CH22103	Chicken, polyclonal	1:1000

doi:10.1371/journal.pone.0146428.t001

Immunohistochemistry

Coronal brain sections were cut in PBS at a thickness of 50 μm using a vibratome (VT1200, Leica Microsystems, Vienna, Austria). Sections including the auditory brainstem and the midbrain were collected and immunofluorescence labeling was performed. Free-floating sections were washed in 0.1 M PBS and blocked for 1 hour at RT in a solution containing 10% normal

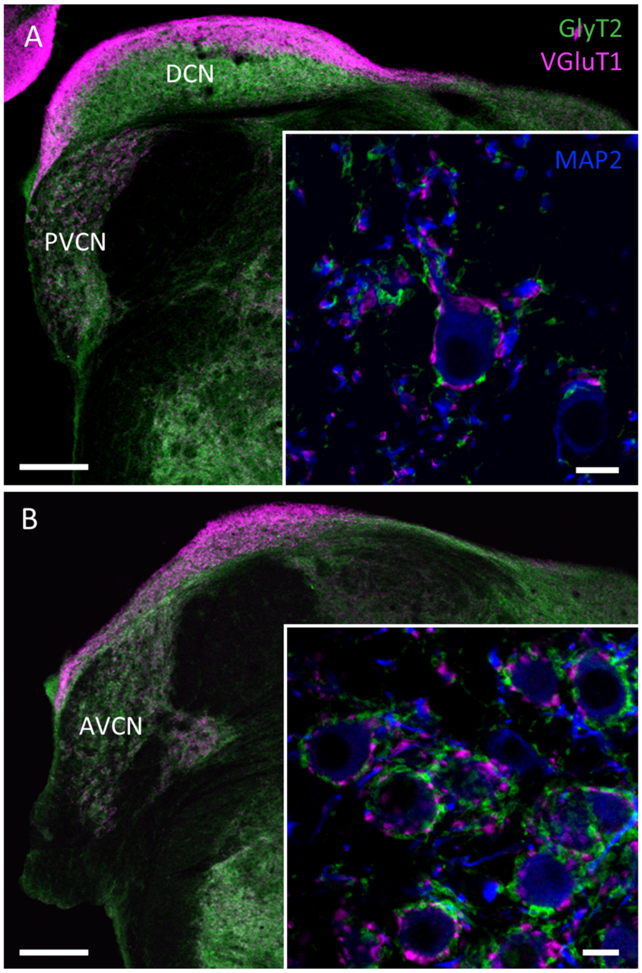

Fig 1. Western blots of hippocampus extracts of mouse and naked mole-rat. Identical band size for VGluT1 (~ 50 kDa) and GlyT2 (~90 kDa) in mouse and naked mole-rat. However, blots for HCN1 detected only the lighter band in naked mole-rat. GAPDH: loading control.

doi:10.1371/journal.pone.0146428.g001

donkey serum diluted in 0.1 M PBS with 0.2% Triton X-100 (PBST). They were incubated for 48 h at 4°C in the primary antisera containing 3% normal donkey serum, 0.2% PBST, rabbit anti-VGluT1 (1:1000), guinea pig anti-GlyT2 (1:1000) and chicken anti-MAP2 (1:1000), diluted in 0.1 M PBS. For HCN1 labeling primary antibodies mouse anti-HCN1 (1:1000) and chicken anti-MAP2 (1:1000) were used. Subsequently, sections were washed several times and incubated for 2 h at RT in secondary fluorescent antibodies Alexa Fluor 555 donkey anti-rabbit (1:500), Cy3 donkey anti-guinea pig (1:250), Alexa Flour 647 donkey anti-chicken (1:150) and Alexa Flour 488 donkey anti-mouse (1:250), respectively. Negative controls for all antibodies were performed by omitting the primary antibody, followed by the protocol as described above. In addition tests for cross-reactivity of primary and secondary antibodies were conducted for each pair of used antibodies. Sections were mounted and cover slipped using a homemade anti-fading mounting media (refractive index in oil: 1.56) [22]

Image Acquisition, Processing and Quantification

Fluorescent micrographs were acquired using a confocal laser scanning microscope (TCS SP8, Leica Microsystems) equipped with a 5x HCX PL FLUOTAR objective (NA 0.15), 20x HC PL APO Imm. Corr. objective (0.75 NA) and a 63x HCX PL APO immersion oil objective (1.4NA). The pinhole was set to 1 Airy unit for each channel. Illumination and detection pathways were separated for each fluorophore and individual color channels were sequentially acquired to avoid bleed-through artifacts. The acquisition settings were adjusted to cover the entire dynamic range of the detectors and remained unchanged during the course of the imaging process. Z-stacks of confocal images were obtained and maximal projections of 4–10 single optical sections were used for high magnification figures. Low magnification photomicrographs (5x and 20x) are always single optical sections. Fiji (http://fiji.sc; Schindelin et al., 2012) and Adobe Photoshop CS6 (Adobe Systems, San Jose, CA) were used to adjust brightness and contrast.

Quantification of HCN1 immunostaining was conducted by densitometry in two naked-mole rats and two mice. Experiments were performed as described above. High magnification images (63x,1.4NA, voxel size 180 nm) of regions containing LSO, MSO and VNLL were quantified using Fiji. At least, the study included five sections spanning the rostrocuadal extent of each nucleus. Mean grey values of each optical section were obtained and subsequently averaged for the entire Z-stack. To compare among cases, the averaged mean gray value was corrected by the subtraction of overall pixel intensity of the unspecific background staining in HCN1-negative areas within the same section. The medial nucleus of the trapezoid body (MNTB) was selected as negative control for LSO and MSO quantification, and the pontine nuclei (PN) for VNLL quantification.

Western Blotting

Two naked mole-rats and two C57Bl/6 mice were used to test antibody specificity in naked mole-rat brains. Animals were decapitated under isoflurane anesthesia. Immediately after, brains were dissected out and samples containing brainstem, cerebellum, hippocampus and inferior colliculus were collected, frozen and stored at -80°C. Brain protein extracts and western blotting were performed as described elsewhere [23]. Brain samples were homogenized in RIPA buffer (10mM TrisHCl pH7.5, 100mM NaCl, 2mM EDTA, 1% NP-40, with proteinase inhibitors). The homogenate was centrifuged at 13,500 rmp for 15 minutes at 4°C and the supernatant was collected. Protein concentration was determined using Bradford method. Brain samples were boiled for 5 min at 95°C in loading buffer. One hundred micrograms of total protein extracts were electrophoresed on 8% sodium dodecyl sulfate (SDS) polyacryl-

amide gels (Laemmli, 1970) by using the mini-PROTEAN III system (Bio-Rad, Hercules, CA) for an hour at 130 V. Gels were subsequently transferred onto PVFD membranes for 1 hour and a half at 75 mA/gel using a semidry blotter (Bio-Rad). The Blots were blocked in TBST-milk (50 mM Tris, pH 7.5, 200 mM NaCl, 0.1% Tween 20, and 5% nonfat dry milk) for 1 hour at RT and further incubated with their corresponding primary anti-serum (1:2000 α-HCN1,1: 4000 α -GlyT2, 1:10000, α -VGluT1). Blots were next incubated with the appropriate secondary antiserum (horseradish peroxidase-conjugated IgG, 1:1000, Pierce) for 2 hours at RT. Bound secondary antibodies were detected using an enhanced chemiluminescence assay (west dura substrate, Pierce) and the chemo-luminescent signal was developed by X-ray films and further scanned. Additionally, blots showing antibody-bound signal was removed with a stripping buffer (Pierce) at 37°C for 30 minutes and further exposed to GAPDH (1:2000, Applied Biosystems) for loading control test.

Results

Naked Mole-Rat Auditory Brainstem Anatomy Based on the Distribution of Excitatory and Inhibitory Inputs

We identified the auditory brainstem nuclei in the naked mole-rat using antibody staining against two presynaptic proteins, namely the α-VGluT1, which is present in a large subset of glutamatergic synaptic endings, and the α-GlyT2, the main transporter in glycinergic presynaptic structures. These transporters are present in the majority of excitatory and inhibitory presynaptic endings in monaural and binaural auditory brainstem nuclei and are therefore well suited to characterize the homology of auditory brainstem nuclei across different species [24–27]. Moreover, the distinct expression pattern of VGluT1 and VGluT2 in auditory brainstem nuclei allows further classifications of auditory input specificity [16,26,28]. The physiological characteristics and the spatial distribution of the excitatory and inhibitory inputs are well characterized in most auditory brainstem nuclei of several different species. We used this additional knowledge to unambiguously identify the auditory brainstem nuclei in the naked mole-rat and correlate them with their function.

Similar to a previous study of naked mole-rat auditory brainstem anatomy [1] we show that despite their relatively poor hearing abilities the major auditory brainstem nuclei are well preserved. On the basis of labelling against glutamatergic and glycinergic inputs, the major subdivisions of the cochlear nucleus (CN) could be clearly identified (Fig 2), and distinct boundaries between the dorsal (DCN) and the ventral (VCN) parts of the CN could be drawn. Fig 2A displays a more caudal coronal section of the CN including the dorsal cochlear nucleus (DCN), where the most superficial layer is highly enriched with VGluT1 and more ventrally the posterior ventral cochlear nucleus (PVCN). Both of these nuclei are similar in structure and size when compared to mice and rats [29,30]. However, unlike in rats a GlyT2-negative area in the dorsal part of the PVCN is missing. This area is generally highly enriched with octopus cells, which lack GlyT2 immunoreactivity [24]. This missing GlyT2 negative area indicates that octopus cells might be functionally changed or even missing in the PVCN of the naked mole-rat. Further magnification reveals of a more ventral part of the PVCN reveals abundant excitatory and inhibitory inputs on a presumed stellate cell, similar to other rodent species (Fig 2A *inset*). Fig 2B displays an overview of a more rostral part of the CN including the anterior ventral cochlear nucleus (AVCN) and a small part of the DCN. Again, this overall distribution of excitatory and inhibitory inputs in the AVCN resembles the one observed in other rodent species. Higher magnification reveals large VGluT1 positive endings around the somata of presumed bushy cells that represent the synaptic inputs from the auditory nerve fibers (Endbulbs of Held) [16,31–33] (Fig 2B *inset*). However, this VGluT1 immunoreactivity around the somata

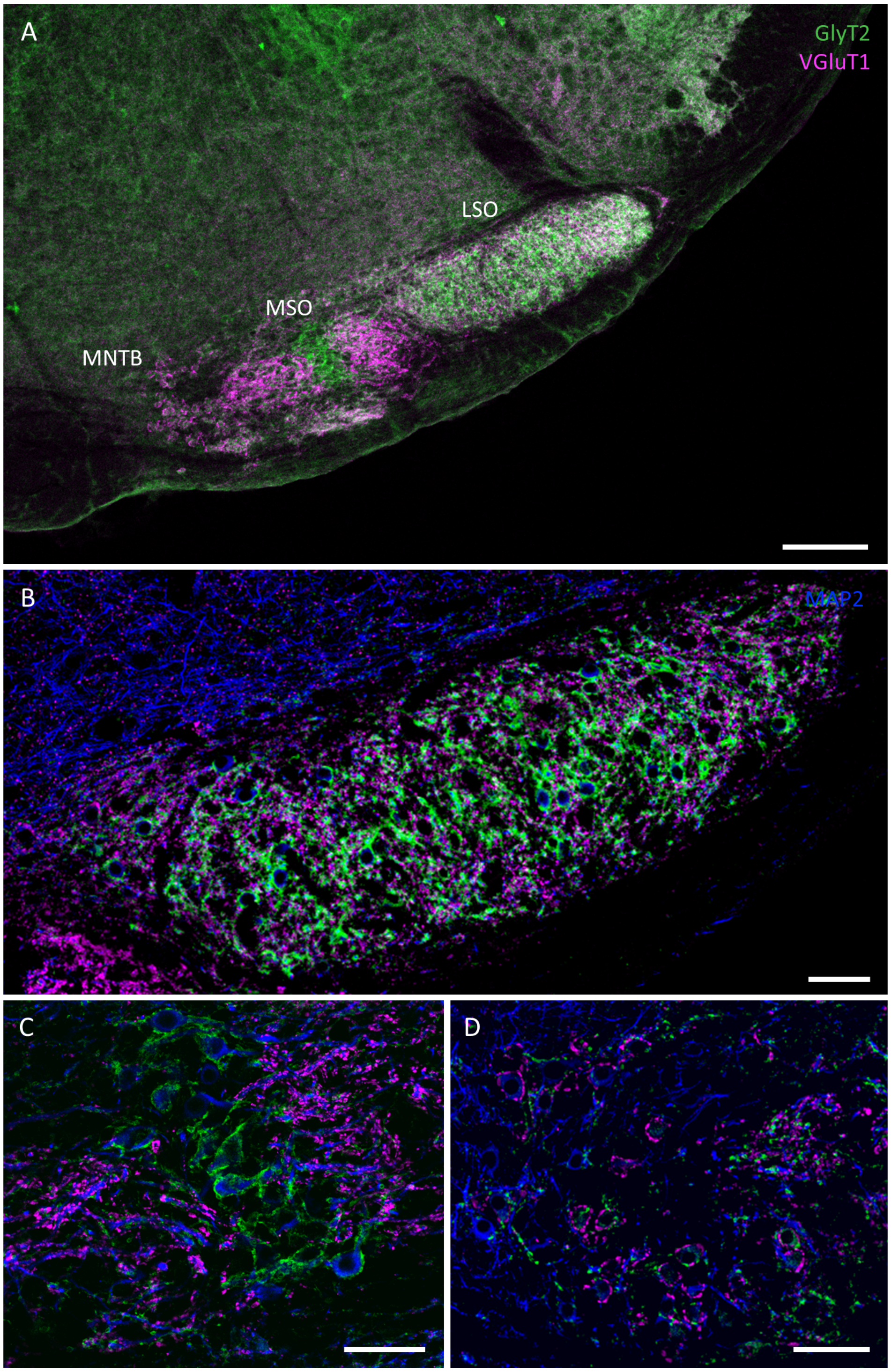

Fig 2. Confocal images illustrating inhibitory (GlyT2, green) and excitatory (VGluT1, magenta) synaptic inputs counterstained with MAP2 (blue) at two interaural levels through the cochlear nucleus (CN) of naked mole-rat. (A) Coronal section showing prominent dorsal (DCN) and posteroventral (PVCN) subdivisions of the CN. Note in the inset the distribution of the inputs apposing a neuron located in the region of the octopus cells. **(B)** Rostral section of the CN including the anteroventral subdivision (AVCN). Note in the inset a detail of synaptic contacting a bushy cell. Scale bars: 200 μm, insets: 10 μm.

doi:10.1371/journal.pone.0146428.g002

and especially in the neuropil seems to be more scattered and scarce compared to the other rodents including guinea pig, rat and mouse [16,34,35], which might indicate less elaborated arborization of the endbulbs of Held. The glycinergic GlyT2 positive endings were virtually distributed around the soma of these neurons, similar to other species [18,36,37].

The superior olivary complex (SOC) is the first site of binaural integration where interaural time and intensity differences (ITDs and IIDs) are analyzed for sound localization in the horizontal plane [38]. Animals with good low frequency hearing (below 1 kHz) use ITDs for sound localization. These ITDs are mainly analyzed by neurons in the medial superior olive (MSO). Animals that lack low-frequency hearing (< 2 kHz) usually rely on IID analysis for localizing sounds. These binaural intensity differences are initially analyzed by neurons in the lateral superior olive (LSO). In the naked mole-rat, the three main SOC nuclei, namely the MSO, the LSO and the medial nucleus of the trapezoid body (MNTB), can be easily identified upon their characteristic shape and their excitatory and inhibitory input pattern as outlined below (Fig 3A). The oval shaped LSO appears to be large and elongated in the medial-lateral axis and GlyT2 and VGluT1 positive endings are uniformly distributed along the medio-lateral axis of the nucleus (Fig 3B). Directly medial to the LSO lies the MSO (Fig 3A). Similar to their arrangement in other low frequency hearing and ITD coding animals [39], these neurons are horizontally layered with their dendrites protruding medially and laterally. They receive pronounced somatic glycinergic inputs and dendritic glutamatergic inputs as depicted by the striped arrangement of GlyT2 and VGluT1 immunoreactivity (Fig 3A and 3C). Taking into account that naked mole-rats are predominantly low frequency hearing animals, the dorso-ventral extent of the MSO is relatively small compared to the LSO. Medial to the MSO, scattered neurons with a diameter of around 10 μm surrounded by large presynaptic VGluT1 positive endings represent the medial nucleus of the trapezoid body (MNTB) (Fig 3A). The VGluT1 positive ring around the somata indicates the existence of a Calyx of Held synapse. These MNTB neurons are only sparsely intermingled within the fiber tract of the trapezoid body (Fig 3C). It is important to note that the superior periolivary nucleus (SPN), which is generally located medial-dorsal from the LSO and typically lacks VGluT1 positive inputs [14] but receives numerous glycinergic inputs, could not be identified (Fig 3A). Since, VGluT1 and GlyT2 positive endings are distributed equally across the LSO it is also unlikely that the SPN and the LSO are fused together. This indicates that SPN neurons are either degenerated in the naked mole-rat brain or lack the typical VGluT2-only positive excitatory inputs as observed in other species. The periolivary nuclei such as the ventral and lateral nucleus of the trapezoid body (VNTB and LNTB) are small (VNTB) or could not be identified (LNTB) with this labeling approach. Both nuclei show prominent VGluT1 and GlyT2 staining in the rat.

Fig 4 depicts the subcellular distribution of VGluT1 and GlyT2 positive endings in the LSO, MSO and MNTB. As typically, MNTB somata are surrounded by large VGluT1 positive terminals, that resemble the Calyx of Held synaptic terminals in other species (Fig 4A). In the LSO the inhibitory GlyT2 positive endings surround the somata whereas the excitatory VGluT1 positive endings are spread throughout the neuropil contacting the small round dendrites that are also positive for MAP2 (Fig 4B). A similar somato-dendritic segregation of glutamatergic and glycinergic inputs can be observed in the MSO (Fig 4C). There GlyT2 positive endings are exclusively found on the somatic membranes, whereas all VGluT1 positive endings contact the medial and lateral dendrites of these neurons.

In mammals the nuclei of the lateral lemniscus receive inputs from the ventral cochlear nucleus and the SOC and provide strong inhibitory glycinergic and GABAergic projections to the inferior colliculus [40–42]. In the naked mole-rat two nuclei of the lateral lemniscus could be clearly distinguished according to their distinct staining pattern of excitatory and inhibitory inputs (Fig 5A). The ventral nucleus of the lateral lemniscus (VNLL) appears as a banana-

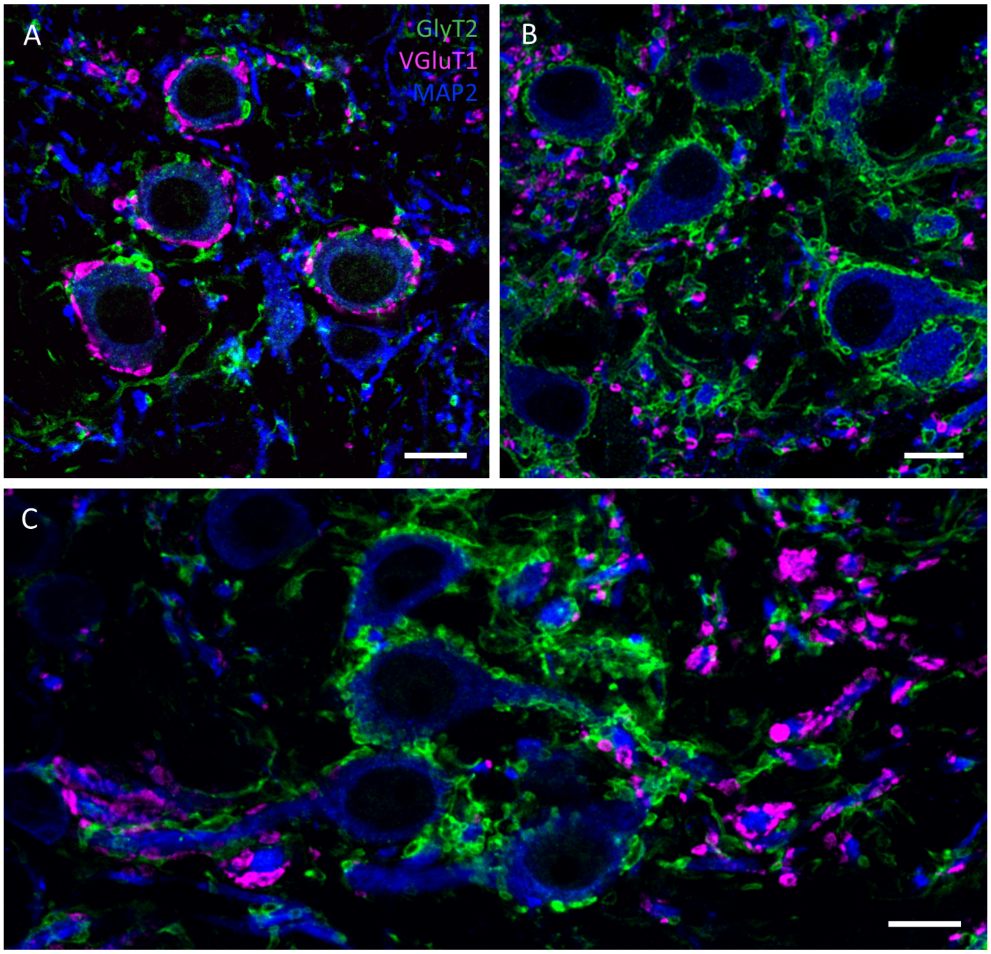

Fig 3. Confocal images showing GlyT2 (green), VGluT1 (magenta), MAP2 (blue) immunostaining in the superior olivary complex (SOC) of the naked mole-rat. (A) overview of the three major SOC nuclei: lateral superior olive (LSO), medial superior olive (MSO), medial nucleus of the trapezoid body (MNTB); **(B)** large and elongated LSO **(C)** small MSO **(D)** MNTB with sparsely distributed neurons. Scale bars: A: 200 μm; B-D: 50 μm.

doi:10.1371/journal.pone.0146428.g003

shaped shaped structure where neurons are intermingled throughout the lemniscal fiber tract. Higher magnification of VNLL neurons depict numerous VGluT1 positive endings around the somata (Fig 5D). Moreover GlyT2 positive endings are located on the somata and the dendrites in the VNLL. In contrast, neurons in the dorsal nucleus of the lateral lemniscus (DNLL) mostly lack VGluT1-positive synaptic terminals (Fig 5A and 5C). This is consistent with the vGluT2 positive projections to the DNLL that mainly originate in the LSO and MSO [26,43,44]. Therefore, mainly GlyT2 positive endings could be observed in the DNLL with our staining protocol.

The inferior colliculus (IC) is a major auditory integration center which receives excitatory and inhibitory inputs from all lower auditory brainstem areas. In the naked mole-rat the IC is well developed with no apparent differences in size and excitatory and inhibitory input pattern to other species (Fig 5A). VGluT1 positive inputs are abundant and mostly localized within the neuropil, whereas GlyT2 positive endings are arranged around the somata of many neurons (Fig 5B). This is in contrast to findings in the rat, where GlyT2 positive endings were mostly found in the neuropil of the IC [24].

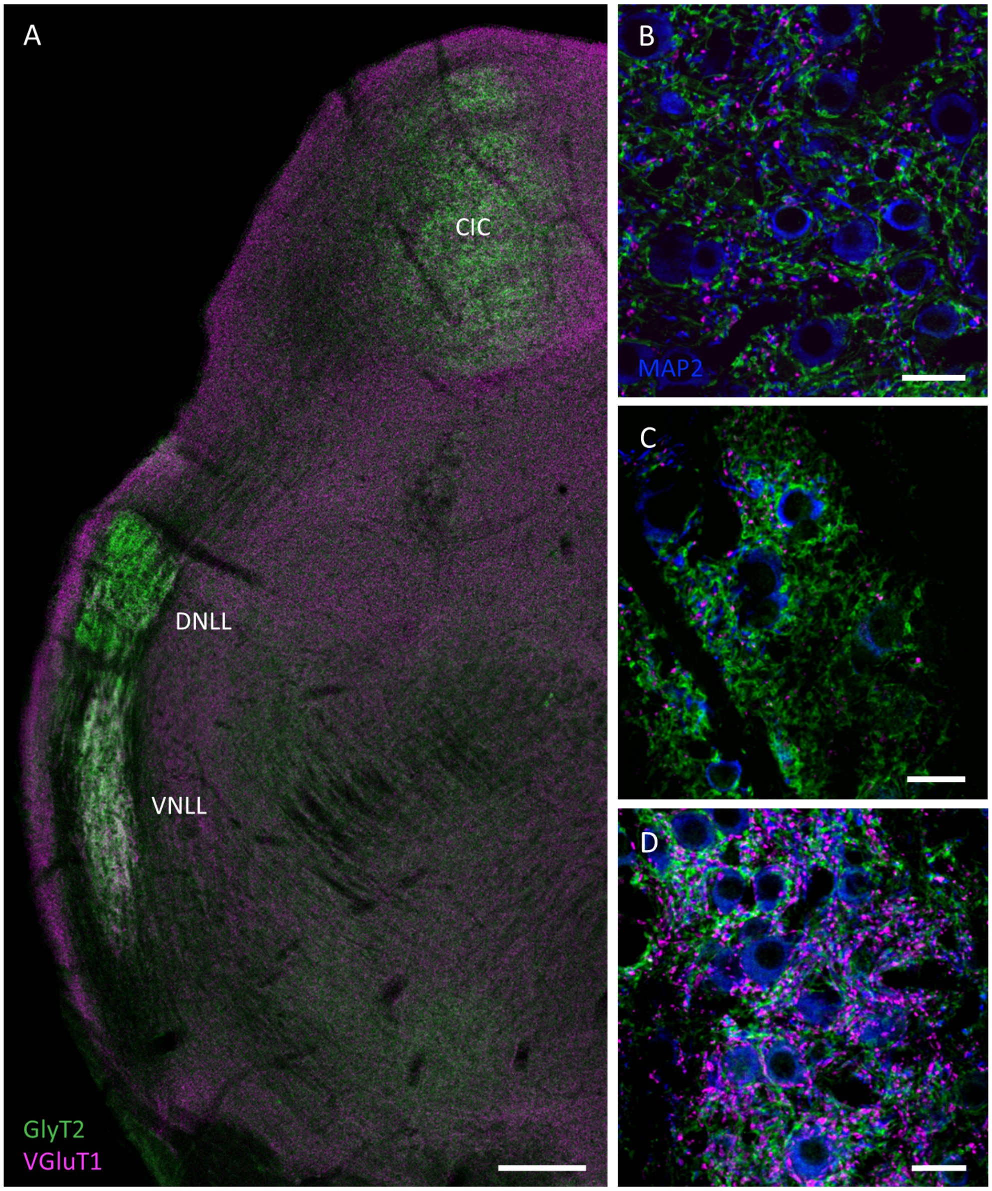

Fig 4. Confocal images depicting GlyT2 (green) and VGluT1 (magenta) positive synaptic inputs contacting with MNTB (A), LSO (B) and MSO (C) neurons (MAP2: blue). (A) Large vGluT1 perisomatic profiles around MNTB neurons suggest calyx-like synaptic structures. **(B)** strong GlyT2 positive endings surround the somata of LSO neurons. while excitatory vGluT1 endings are located in the neuropil. **(C)** MSO neurons with GlyT2 positive terminals mainly around the somata and vGluT1 positive endings on the dendrites of the neurons. Scale bars: 10 μm.

doi:10.1371/journal.pone.0146428.g004

Distribution of HCN1 Channels in the Auditory Brainstem of NRMs

To find out whether neurons in the monaural and binaural auditory brainstem regions also exhibit the fast integrative membrane properties essential for processing of temporal information of sounds, we immunolabelled naked mole-rat auditory brainstem sections against the voltage-gated HCN1 channel. HCN1 channels are already open at the resting membrane potential, thereby contributing to the low membrane time constants required for the precise temporal integration of excitatory and inhibitory inputs [12,21]. VGluT1-immunolabeling was

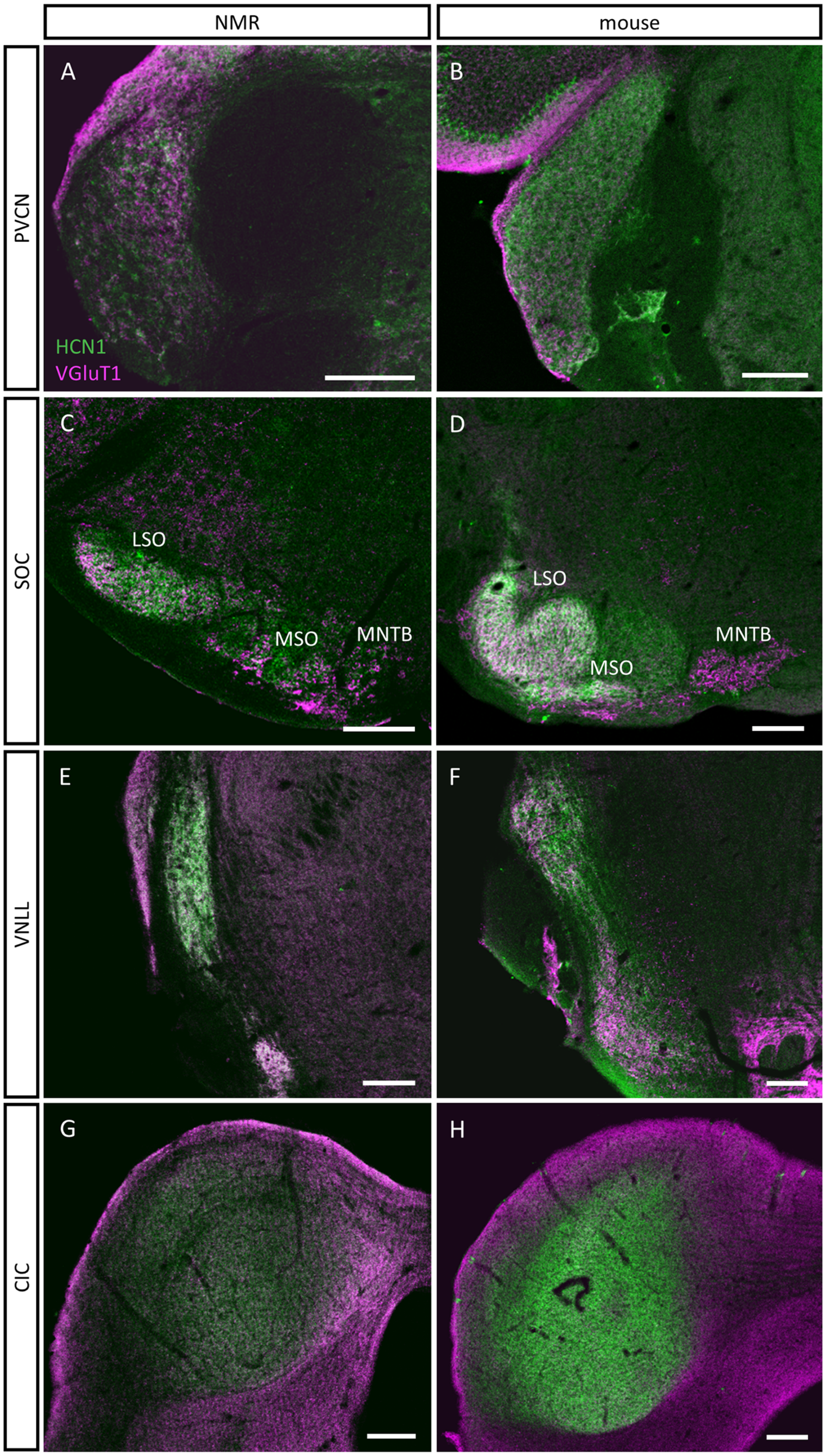

Fig 5. Confocal images showing GlyT2 (green), vGluT1 (magenta), MAP2 (blue) immunostaining in coronal sections containing the nuclei of the lateral lemniscus (LL) and the inferior colliculus (IC) of the naked mole-rat. (A) overview showing a conspicuous central nucleus of the IC (CIC), dorsal nucleus of the LL (DNLL) and ventral nucleus of the LL (VNLL). **(B)** GlyT2 positive endings around the somata and vGLUT1 positive endings in the neuropil are observed in the CIC. **(C)** Abundant GlyT2 positive endings and

only few vGluT1 positive endings in the DNLL. **(D)** VNLL neurons show strong vGluT1 perisomatic profiles. Scale bars: A: 200 μm; B-D: 20 μm.

doi:10.1371/journal.pone.0146428.g005

used as a counterstain to identify the regions of interest. Distribution and intensity of HCN1 immunolabelling in the naked mole-rat was directly compared to mouse brain sections, where also physiological measurements of the hyperpolarization-activated current (Ih) have been obtained [45]. This allows us to also make assumptions of Ih density in naked mole-rat neurons.

In general HCN1 immunostaining was faint and fuzzy in almost the entire brainstem and midbrain of the naked mole-rat when compared to mouse tissue, with only one exception the VNLL.

Overviews of HCN1 and VGluT1 immunostainings show that HCN1 immunoreactivity was faint in the naked mole-rat PVCN, but prominent in the mouse (Fig 6A and 6B). As previously shown, intense HCN1 immunoreactivity was observed in the LSO and MSO of the mouse, but not in the MNTB (Fig 6D) [45]. In stark contrast, naked mole-rat HCN1 immunoreactivity was again considerably fainter in the entire SOC including the LSO and the MSO (Fig 6C). To our surprise, this was different in the VNLL. There, HCN1-immunoreactivity was intense in the naked mole-rat and comparable to the HCN1 intensity observed in the mouse VNLL (Fig 6E and 6F). In the inferior colliculus HCN1 immunoreactivity was again much lower in the naked mole-rat, when compared to the mouse.

High magnification of HCN1 immunoreactivity in the respective regions illustrate that in the LSO (Fig 7A) and the MSO (Fig 7C) HCN1 immunopositive punctae are scarce and mostly located in the cytosol and not on the membranes of the respective neurons. In contrast, HCN1 positive punctae densely label the somatic and dendritic membranes of LSO and MSO neurons in mice (Fig 7B and 7D). In contrast to this, naked mole-rat VNLL neurons display intense and punctate HCN1 immunolabelling on the somatic and dendritic membranes very similar to the mouse VNLL neurons (Fig 7E and 7F). Quantification of overall—membrane bound and cytosolic—HCN1 immunoreactivity in the respective nuclei shows that overall mean grey value (mgv) was approximately twice as large in the mouse LSO compared to the naked mole-rat LSO (NMR mgv 58% of mouse mgv). This difference in HCN1 immunoreactivity was even larger between in the naked mole-rat and mouse MSO (NMR mgv 34% of mouse mgv). On the contrary, overall immunoreactivity as measured was very similar in the VNLL in both species (NMR mgv 88% of mouse mgv) (Fig 7G). This suggests that in the naked mole-rat auditory brainstem only VNLL neurons have a large density of functional HCN1 channels and might therefore exhibit membrane properties suited for analyzing rapidly changing inputs.

To investigate whether the HCN1 expression profile differs between the naked mole-rat and the mouse we performed a western-blot on samples from several brain areas including hippocampus, brainstem and inferior colliculus. In all analyzed mouse samples, the western blots gave rise to two distinct bands (Fig 1). We observed a prominent band of ~125 kD as well as a fainter one of ~110 kD. In murine brain tissue HCN1 is found predominantly in its glycosylated isoform, which is important for the insertion of these channels into the membrane [46]. However, naked mole-rat western blots showed only the lighter band, indicating lack of the glycosylated isoform of HCN1 channels in naked mole-rats, which might prevent a proper insertion of the channel into the membrane.

Discussion

The present study aimed to identify and characterize the different nuclei of the naked mole-rat auditory brainstem and relate these finding to the specialized auditory environment naked

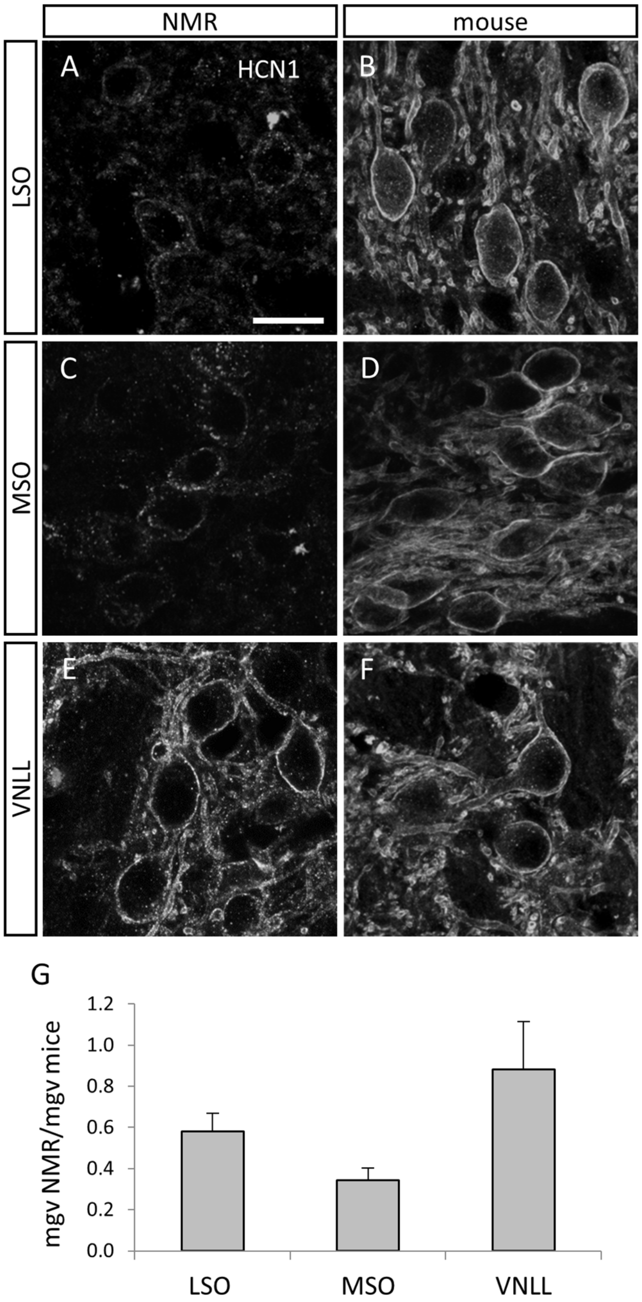

Fig 6. Confocal images depicting HCN1 (green) and vGluT1 (magenta) immunostaining in the PVCN (A,B), the SOC (C,D), the VNLL (E,F) and the inferior colliculus (G,H) of naked mole-rat (NMR) (left column) and mouse (right column). HCN1 immunolabelling is diffuse and faint in the PVCN, SOC and CIC nuclei of the naked mole-rat **(A, C, G)**, but more intense in the VNLL **(E)**; in contrast, strong HCN1 immunolabelling is present in the PVCN, the MSO and LSO, the VNLL and the IC of mouse **(B, D, F, H)**. Scale bars: 200 μm.

doi:10.1371/journal.pone.0146428.g006

mole-rats live in. We used three different anatomical marker, namely the vesicular glutamate transporter VGluT1, the glycine transporter GlyT2 and the hyperpolarization activated channel HCN1. The distribution for each of these markers is well described in the rodent auditory brainstem and can be tightly correlated to their underlying physiology. Based on the

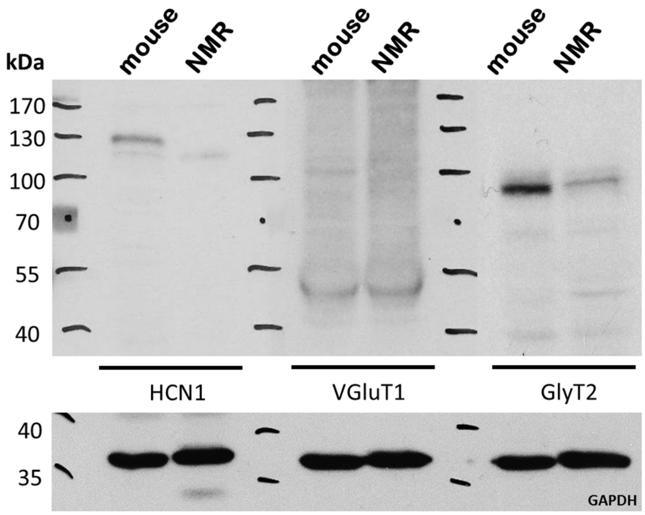

Fig 7. Confocal images illustrating HCN1 immunostaining in the LSO (A,B), MSO (C,D) and VNLL (E,F) of NMR (left column) and mouse (right column). In the naked mole-rat, diffuse and somatic staining against HCN1 was observed in LSO **(A)** and MSO **(C)** neurons. In contrast, intense membrane bound HCN1 immunolabeling was found in the equivalent auditory brainstem nuclei of the mouse: LSO neurons **(B)** and MSO neurons **(D)**. Comparably strong and membrane bound HCN1 immunostaining in VNLL neurons of the naked mole-rat **(E)** and mouse **(F)**. Ratio of averaged and background-subtracted mean grey value (mgv) of HCN1 immunolabelling for the LSO, MSO and VNLL of naked mole rats (n = 2) and mice (n = 2). Error bars are standard error of mean **(G)** Scale bar: 20 μm.

doi:10.1371/journal.pone.0146428.g007

distribution of VGluT1 and GlyT2 in naked mole-rats we identified all the major nuclei in the auditory brainstem with one exception, the SPN. The divisions of the cochlear nucleus, the lateral lemniscus and the inferior colliculus were similar in size and input distribution to other rodents such as rats, gerbils and mice [26,30,47]. In contrast, the binaural nuclei of the SOC were notably altered in structure and size. Neurons in the MNTB were reduced, the MSO was well defined but relatively small, whereas the LSO appeared strikingly enlarged. Most interestingly, the SPN could not be identified with our methods. A second striking results of our experiments was that the HCN1 channel, which is robustly expressed in rodent auditory brainstem neurons that integrate excitatory and inhibitory inputs on a rapid time scale, is faint and cytosolic in the naked mole-rat. This was true for neurons in the PVCN and principal neurons in the LSO and MSO. Only monaural VNLL neurons showed intense and membrane bound HCN1 immunoreactivity in the naked mole-rat, comparable to the mouse.

The Superior Olivary Complex of the Naked Mole-Rat: An Adaptation to Underground Living

As an adaptation to their specialized acoustic environment naked mole-rats seem to have poor sound localization abilities with minimal audible angels that are almost 10-fold larger compared to other rodent species [1]. Given this, it is even more surprising that in the naked mole-rat the two major nuclei analyzing ITDs and IIDs, namely the LSO and the MSO, are large, well defined and receive spatially defined excitatory and inhibitory inputs. Naked mole-rats hear best between 125 to 8000 Hz [1] indicating that these animals could employ ITDs for sound localization. This is corroborated by our finding that the MSO neurons, that analyze ITDs based on the coincidence of the respective inputs from the two ears, display input segregation with excitatory inputs contacting the lateral and medial dendrites and the inhibitory inputs contacting the somatic membrane. A similar segregation of excitatory and inhibitory

synapses was observed in gerbils, that use ITDs of pure tone for sound localization. In high frequency-hearing animals, such as bats and rats, which do not rely on pure tone ITD-analysis, excitatory and inhibitory synapses on MSO neurons are more intermingled [48].

The MNTB is the main inhibitory input to the MSO and LSO [38,49]. We found that the MNTB neurons seem to be reduced and more loosely distributed in the naked mole-rat when compared to gerbils and rats [50,51]. Nevertheless, both MSO and LSO neurons receive numerous and well defined glycinergic endings. This is similar in a mouse model (Egr2; En1CKO), where MNTB neurons are genetically deleted. In this mouse LSO neurons still receive glycinergic inputs, however, with an altered subunit composition and significantly prolonged synaptic time constants [52,53]. Also LNTB neurons, that provide an additional ipsilateral glycinergic input to LSO and MSO neurons [54,55] are preserved and might compensate for the lack of MNTB neurons. This is not possible in the naked mole-rat, where LNTB neurons are scarce or even absent. One possibility is that the pronounced glycinergic inputs in the LSO and MSO originate from few MNTB neurons with highly ramified axons. But also glycinergic inputs from additional sources could project to the MSO and LSO neurons.

In our naked mole-rat sections the LSO is strikingly large and elongated. In a previous study, in which auditory brainstem nuclei were classified on the basis of Nissl staining, the LSO was only approximately about half the diameter compared to our study [1]. One possible explanation is a different rostro-caudal plane of the section. Alternatively, variances in brain anatomy between various naked mole-rat colonies cannot be excluded. Since the SPN could not be detected in our sections we also speculate that the large and elongated LSO in our naked mole-rat sections could actually derive from a merged LSO and SPN. However, the uniform distribution of VGluT1, which is not expressed in the SPN of mice [14], along the entire medio-lateral extent of this nucleus speaks against this hypothesis. Why do naked mole-rats possess such a large LSO when their sound localization abilities are underdeveloped? Naked mole-rats use a large repertoire of communication signals neurons which emphasizes the importance of sound pattern analysis. Thus, it is possible that both the LSO and MSO circuits not only process binaural acoustic cues, but are also involved in monaural sound pattern analysis as previously suggested [56–58]. The lack of a classical SPN nucleus, which is also considered to process sound patterns [59,60], supports this hypothesis.

Lack of HCN1 Channels in Binaural Auditory Brainstem Neurons: Consequences for Sound Localization

Surprisingly, HCN1- immunolabelling was faint in all naked mole-rat auditory brainstem nuclei except for the VNLL. Moreover, HCN1-immunoreactive punctae were mostly located in the cytosol and not, as expected, localized to the membranes of neurons. This was especially apparent when naked mole-rat HCN1 immunostaining was compared to mice, where the same staining procedure produced strong membrane bound HCN1 immunolabelling in the octopus area of the PVCN, the LSO and the VNLL, as previously described [17,45]. The strong labeling for HCN1 in the naked mole-rat VNLL indicates that the relative lack of labeling in other nuclei in this species is not due to a problem with antibody binding.

HCN channels are homomeric or heteromeric, pentameric structures formed by the HCN channel isoforms HCN1-4 [61]. HCN1 subtype channels have the fastest activation time constants and the most depolarized half-activation voltage. Therefore a large proportion of the HCN1 subtype channels are open around the resting membrane potential of neurons contributing to fast membrane time constants. High levels of HCN1 channels are found in the binaural nuclei of the auditory brainstem of rodents and birds [12,17,62]. In these neurons blocking of Ih hyperpolarizes the neurons, increases membrane time constants, precludes temporal

summation of inputs and widens the window for coincidence detection of inputs [12,21]. Together with Kv1 channels, a low-voltage activated potassium channels, these ion channels enable the neurons to precisely integrate excitatory and inhibitory synaptic currents [12,63,64].

What might a lack of HCN1 channels mean for the functional integration of synaptic inputs and the detection of coincident inputs? Pharmacological blockade of Ih in gerbil and bird ITD detection circuit, namely the MSO and the nucleus laminaris, widens the time window for the coincidence detection of binaural inputs [62,65]. Moreover, a selective knockout of HCN1 channels increases temporal summation of inputs and reduces the faithful transmission of inputs with short intervals in the LSO [66]. We therefore speculate that a lack of HCN1 channels in the naked mole-rat binaural auditory brainstem neurons profoundly decreases the temporal resolution of binaural input integration and deteriorates the analysis of ITDs to localize sounds. Indeed naked mole-rats need sound pulses up to several hundred milliseconds to adequately localize a sound [1], still their minimal audible angle remaining several-fold larger compared to other rodent species. Ultimately only physiological experiments can unravel the functional set-up of these auditory brainstem nuclei.

It is possible that in the naked mole-rat HCN1 channels are functionally replaced by other HCN channel subtypes. In the rodent auditory brainstem mainly HCN4 and some HCN2 subunits are present apart from HCN1 [12]. However, in the auditory brainstem these subunits form functional heteromers with HCN1 channels [12]. Homomeric channels of these subtypes would have much slower activation time constants and a more hyperpolarized voltage dependence, and are thus less suitable to lower membrane time constants at the resting membrane potential.

Our western-blot suggests that HCN1 channels in the naked mole-rats are weakly expressed, but mainly in the non-glycosylated form, thus preventing the insertion of functional channels in the membrane [46]. This is corroborated by the cytosolic location of HCN1 immunoreactivity observed in LSO and MSO neurons in our sections. Why this is different in the VNLL of the naked mole-rat, where membrane bound HCN1 channels are abundant, is unclear. Functionally, we know that VNLL neurons receive excitatory inputs from octopus cells of the PVCN and inhibitory inputs from the MNTB [40,67]. Integration of these excitatory and inhibitory inputs leads to the temporally highly precise responses of these neurons to structured sounds which is considered to be important for the processing of temporal sounds patterns [68,69]. Thus, membrane bound and functional HCN1 channels might contribute to the analysis of the complex and rapidly fluctuating temporal patterns of naked mole-rats communication sounds.

As mentioned above HCN1 together with Kv1 channels renders neuronal membranes leaky and very permeable for ions already around the resting membrane potential. As a consequence, ATP-consuming ionic pumps need to be continuously active to maintain the ionic gradients across the membrane [70]. Since the operation of ionic pumps uses a large fraction of the total energy consumption of the body, functional HCN1 channels might have been lost in the majority of neurons in the brain to reduce energy consumption in the naked mole-rat. This evolutionary adaptation could be especially important due to the oxygen deprived environment naked mole-rats live in.

Conclusions and Functional Implications for Hearing in Naked Mole Rats

Taken together naked mole-rats possess a highly distinct auditory brainstem with a large LSO but few MNTB neurons, a circuit, whose function remains to be shown. . . Remarkably, the VNLL is the only nucleus in the entire brainstem, where labelling against the HCN1 channel is observed in the membrane, suggesting a particular role of this nucleus for the processing of temporal sound information, as for example communication signals.

Our anatomical data support a previous finding that naked mole-rats have poor sound localization ability [1]. That study also showed that naked mole-rats have generally elevated auditory thresholds compared to surface dwelling mammals. However, elevated thresholds might be less of a hindrance for audio-vocal communication in the NMR's subterranean niche compared to many surface environments. This is because in naked mole-rat burrows is relatively low and naked-mole rat vocalize at frequencies that propagate well [71]. Our anatomical data suggest that despite elevated hearing thresholds, naked mole rat auditory brainstem structures may be well suited for processing the complex vocal repertoire of this highly social [72] and highly vocal species [9,73].

Acknowledgments

We thank Gary Lewin for providing the naked mole-rats from his colony at the Max-Delbrück-Center in Berlin-Buch. This work was supported by DFG grant SPP1608 (U.K.), NeuroCure (U.K.) and a German Excellence Initiative and the Marie Curie Program of the European Commission—DRS postdoctoral fellowhip (E.G.P).

Author Contributions

Conceived and designed the experiments: EGP TP UK. Performed the experiments: NG EGP DO. Analyzed the data: NG EGP UK. Wrote the paper: NG EGP UK.

References

1. Heffner RS, Heffner HE. Degenerate hearing and sound localization in naked mole rats (Heterocephalus glaber), with an overview of central auditory structures. J Comp Neurol. 1993; 331: 418–433. doi: 10.1002/cne.903310311 PMID: 8514919
2. Crish SD, Dengler-Crish CM, Catania KC. Central visual system of the naked mole-rat (Heterocephalus glaber). Anat Rec A Discov Mol Cell Evol Biol. 2006; 288A: 205–212. doi: 10.1002/ar.a.20288
3. Park TJ, Lu Y, Jüttner R, Smith ESJ, Hu J, Brand A, et al. Selective Inflammatory Pain Insensitivity in the African Naked Mole-Rat (Heterocephalus glaber). PLoS Biol. 2008; 6: e13. doi: 10.1371/journal.pbio.0060013 PMID: 18232734
4. Smith ESJ, Omerbašić D, Lechner SG, Anirudhan G, Lapatsina L, Lewin GR. The Molecular Basis of Acid Insensitivity in the African Naked Mole-Rat. Science. 2011; 334: 1557–1560. doi: 10.1126/science.1213760 PMID: 22174253
5. Fang X, Seim I, Huang Z, Gerashchenko MV, Xiong Z, Turanov AA, et al. Adaptations to a Subterranean Environment and Longevity Revealed by the Analysis of Mole Rat Genomes. Cell Rep. 2014; doi: 10.1016/j.celrep.2014.07.030
6. Lange S, Burda H, Wegner RE, Dammann P, Begall S, Kawalika M. Living in a "stethoscope": burrow-acoustics promote auditory specializations in subterranean rodents. Naturwissenschaften. 2007; 94: 134–138. doi: 10.1007/s00114-006-0168-0 PMID: 17119910
7. Heffner RS, Heffner HE. Hearing and sound localization in blind mole rats (Spalax ehrenbergi). Hear Res. 1992; 62: 206–216. doi: 10.1016/0378-5955(92)90188-S PMID: 1429264
8. Lacey EA, Patton JL, Cameron GN. Life Underground: The Biology of Subterranean Rodents. University of Chicago Press; 2000.
9. Sherman PW, Jarvis JUM, Alexander RD. The Biology of the Naked Mole-rat. Princeton University Press; 1991.
10. Credner S, Burda H, Ludescher F. Acoustic communication underground: vocalization characteristics in subterranean social mole-rats (Cryptomys sp., Bathyergidae). J Comp Physiol A. 1997; 180: 245–255. doi: 10.1007/s003590050045 PMID: 9048440
11. Koch U, Braun M, Kapfer C, Grothe B. Distribution of HCN1 and HCN2 in rat auditory brainstem nuclei. Eur J Neurosci. 2004; 20: 79–91. doi: 10.1111/j.0953-816X.2004.03456.x PMID: 15245481
12. Khurana S, Liu Z, Lewis AS, Rosa K, Chetkovich D, Golding NL. An Essential Role for Modulation of Hyperpolarization-Activated Current in the Development of Binaural Temporal Precision. J Neurosci. 2012; 32: 2814–2823. doi: 10.1523/JNEUROSCI.3882-11.2012 PMID: 22357864

13. Wojcik SM, Rhee JS, Herzog E, Sigler A, Jahn R, Takamori S, et al. An essential role for vesicular glutamate transporter 1 (VGLUT1) in postnatal development and control of quantal size. Proc Natl Acad Sci U S A. 2004; 101: 7158–7163. doi: 10.1073/pnas.0401764101 PMID: 15103023

14. Blaesse P, Ehrhardt S, Friauf E, Nothwang HG. Developmental pattern of three vesicular glutamate transporters in the rat superior olivary complex. Cell Tissue Res. 2005; 320: 33–50. doi: 10.1007/s00441-004-1054-8 PMID: 15714284

15. Gillespie DC, Kim G, Kandler K. Inhibitory synapses in the developing auditory system are glutamatergic. Nat Neurosci. 2005; 8: 332–338. doi: 10.1038/nn1397 PMID: 15746915

16. Zhou J, Nannapaneni N, Shore S. Vessicular glutamate transporters 1 and 2 are differentially associated with auditory nerve and spinal trigeminal inputs to the cochlear nucleus. J Comp Neurol. 2007; 500: 777–787. doi: 10.1002/cne.21208 PMID: 17154258

17. Koch U, Braun M, Kapfer C, Grothe B. Distribution of HCN1 and HCN2 in rat auditory brainstem nuclei. Eur J Neurosci. 2004; 20: 79–91. doi: 10.1111/j.0953-816X.2004.03456.x PMID: 15245481

18. Caminos E, Garcia-Pino E, Martinez-Galan JR, Juiz JM. The potassium channel KCNQ5/Kv7.5 is localized in synaptic endings of auditory brainstem nuclei of the rat. J Comp Neurol. 2007; 505: 363–378. doi: 10.1002/cne.21497 PMID: 17912742

19. Couchman K, Grothe B, Felmy F. Medial superior olivary neurons receive surprisingly few excitatory and inhibitory inputs with balanced strength and short-term dynamics. J Neurosci. 2010; 30: 17111–17121. doi: 10.1523/JNEUROSCI.1760-10.2010 PMID: 21159981

20. Hassfurth B, Grothe B, Koch U. The Mammalian Interaural Time Difference Detection Circuit Is Differentially Controlled by GABAB Receptors during Development. J Neurosci. 2010; 30: 9715–9727. doi: 10.1523/JNEUROSCI.1552-10.2010 PMID: 20660254

21. Baumann V, Lehnert S, Leibold C, Koch U. Tonotopic organization of the hyperpolarization-activated current (Ih) in the mammalian medial superior olive. Front Neural Circuits. 2013; 7: 117. doi: 10.3389/fncir.2013.00117 PMID: 23874271

22. Indig FE, Diaz-Gonzalez F, Ginsberg MH. Analysis of the tetraspanin CD9-integrin alphaIIbbeta3 (GPIIb-IIIa) complex in platelet membranes and transfected cells. Biochem J. 1997; 327: 291–298. PMID: 9355765

23. Preußner M, Wilhelmi I, Schultz A- S, Finkernagel F, Michel M, Möröy T, et al. Rhythmic U2af26 Alternative Splicing Controls PERIOD1 Stability and the Circadian Clock in Mice. Mol Cell. 2014; 54: 651–662. doi: 10.1016/j.molcel.2014.04.015 PMID: 24837677

24. Friauf E, Aragón C, Löhrke S, Westenfelder B, Zafra F. Developmental expression of the glycine transporter GLYT2 in the auditory system of rats suggests involvement in synapse maturation. J Comp Neurol. 1999; 412: 17–37. doi: 10.1002/(SICI)1096-9861(19990913)412:1<17::AID-CNE2>3.0.CO;2-E PMID: 10440707

25. Billups B. Colocalization of vesicular glutamate transporters in the rat superior olivary complex. Neurosci Lett. 2005; 382: 66–70. doi: 10.1016/j.neulet.2005.02.071 PMID: 15911123

26. Ito T, Bishop DC, Oliver DL. Expression of glutamate and inhibitory amino acid vesicular transporters in the rodent auditory brainstem. J Comp Neurol. 2011; 519: 316–340. doi: 10.1002/cne.22521 PMID: 21165977

27. Couchman K, Grothe B, Felmy F. Functional localization of neurotransmitter receptors and synaptic inputs to mature neurons of the medial superior olive. J Neurophysiol. 2012; 107: 1186–1198. doi: 10.1152/jn.00586.2011 PMID: 22131383

28. Ito T, Oliver DL. Origins of glutamatergic terminals in the inferior colliculus identified by retrograde transport and expression of VGLUT1 and VGLUT2 genes. Front Neuroanat. 2010; 4: 135. doi: 10.3389/fnana.2010.00135 PMID: 21048892

29. Muniak MA, Rivas A, Montey KL, May BJ, Francis HW, Ryugo DK. 3D model of frequency representation in the cochlear nucleus of the CBA/J mouse. J Comp Neurol. 2013; 521: 1510–1532. doi: 10.1002/cne.23238 PMID: 23047723

30. Malmierca MS. The structure and physiology of the rat auditory system: an overview. Int Rev Neurobiol. 2003; 56: 147–211. PMID: 14696313

31. Ryugo DK, Fekete DM. Morphology of primary axosomatic endings in the anteroventral cochlear nucleus of the cat: a study of the endbulbs of Held. J Comp Neurol. 1982; 210: 239–257. doi: 10.1002/cne.902100304 PMID: 7142440

32. Benson CG, Gross JS, Suneja SK, Potashner SJ. Synaptophysin immunoreactivity in the cochlear nucleus after unilateral cochlear or ossicular removal. Synapse. 1997; 25: 243–257. doi: 10.1002/(SICI)1098-2396(199703)25:3<243::AID-SYN3>3.0.CO;2-B PMID: 9068122

33. Limb CJ, Ryugo DK. Development of Primary Axosomatic Endings in the Anteroventral Cochlear Nucleus of Mice. JARO J Assoc Res Otolaryngol. 2000; 1: 103–119. doi: 10.1007/s101620010032 PMID: 11545139

34. Gomez-Nieto R, Rubio ME. A bushy cell network in the rat ventral cochlear nucleus. J Comp Neurol. 2009; 516: 241–263. doi: 10.1002/cne.22139 PMID: 19634178

35. Strenzke N, Chanda S, Kopp-Scheinpflug C, Khimich D, Reim K, Bulankina AV, et al. Complexin-I Is Required for High-Fidelity Transmission at the Endbulb of Held Auditory Synapse. J Neurosci. 2009; 29: 7991–8004. doi: 10.1523/JNEUROSCI.0632-09.2009 PMID: 19553439

36. Wenthold RJ, Parakkal MH, Oberdorfer MD, Altschuler RA. Glycine receptor immunoreactivity in the ventral cochlear nucleus of the guinea pig. J Comp Neurol. 1988; 276: 423–435. doi: 10.1002/cne.902760307 PMID: 2848063

37. Mahendrasingam S, Wallam CA, Hackney CM. An immunogold investigation of the relationship between the amino acids GABA and glycine and their transporters in terminals in the guinea-pig anteroventral cochlear nucleus. Brain Res. 2000; 887: 477–481. doi: 10.1016/S0006-8993(00)03054-7 PMID: 11134646

38. Grothe B, Pecka M, McAlpine D. Mechanisms of sound localization in mammals. Physiol Rev. 2010; 90: 983–1012. doi: 10.1152/physrev.00026.2009 PMID: 20664077

39. Rautenberg PL, Grothe B, Felmy F. Quantification of the three-dimensional morphology of coincidence detector neurons in the medial superior olive of gerbils during late postnatal development. J Comp Neurol. 2009; 517: 385–396. doi: 10.1002/cne.22166 PMID: 19760600

40. Schofield BR, Cant NB. Ventral nucleus of the lateral lemniscus in guinea pigs: Cytoarchitecture and inputs from the cochlear nucleus. J Comp Neurol. 1997; 379: 363–385. doi: 10.1002/(SICI)1096-9861(19970317)379:3<363::AID-CNE4>3.0.CO;2-1 PMID: 9067830

41. Pollak GD, Burger RM, Klug A. Dissecting the circuitry of the auditory system. Trends Neurosci. 2003; 26: 33–39. doi: 10.1016/S0166-2236(02)00009-7 PMID: 12495861

42. Kelly JB, van Adel BA, Ito M. Anatomical projections of the nuclei of the lateral lemniscus in the albino rat (rattus norvegicus). J Comp Neurol. 2009; 512: 573–593. doi: 10.1002/cne.21929 PMID: 19034907

43. Glendenning KK, Brusno-Bechtold JK, Thompson GC, Masterton RB. Ascending auditory afferents to the nuclei of the lateral leminscus. J Comp Neurol. 1981; 197: 673–703. doi: 10.1002/cne.901970409 PMID: 7229133

44. Oliver DL. Ascending efferent projections of the superior olivary complex. Microsc Res Tech. 2000; 51: 355–363. doi: 10.1002/1097-0029(20001115)51:4<355::AID-JEMT5>3.0.CO;2-J PMID: 11071719

45. Leao KE, Leao RN, Sun H, Fyffe REW, Walmsley B. Hyperpolarization-activated currents are differentially expressed in mice brainstem auditory nuclei. J Physiol. 2006; 576: 849–864. doi: 10.1113/jphysiol.2006.114702 PMID: 16916913

46. Much B, Wahl-Schott C, Zong X, Schneider A, Baumann L, Moosmang S, et al. Role of Subunit Heteromerization and N-Linked Glycosylation in the Formation of Functional Hyperpolarization-activated Cyclic Nucleotide-gated Channels. J Biol Chem. 2003; 278: 43781–43786. doi: 10.1074/jbc.M306958200 PMID: 12928435

47. Mylius J, Brosch M, Scheich H, Budinger E. Subcortical auditory structures in the mongolian gerbil: I. Golgi architecture. J Comp Neurol. 2013; 521: 1289–1321. doi: 10.1002/cne.23232 PMID: 23047461

48. Kapfer C, Seidl AH, Schweizer H, Grothe B. Experience-dependent refinement of inhibitory inputs to auditory coincidence-detector neurons. Nat Neurosci. 2002; 5: 247–253. doi: 10.1038/nn810 PMID: 11850629

49. Kandler K. Activity-dependent organization of inhibitory circuits: lessons from the auditory system. Curr Opin Neurobiol. 2004; 14: 96–104. doi: 10.1016/j.conb.2004.01.017 PMID: 15018944

50. Löhrke S, Friauf E. Developmental distribution of the glutamate receptor subunits KA2, GluR6/7, and δ1/2 in the rat medial nucleus of the trapezoid body. Cell Tissue Res. 2002; 308: 19–33. doi: 10.1007/s00441-002-0533-z PMID: 12012203

51. Ford MC, Grothe B, Klug A. Fenestration of the calyx of held occurs sequentially along the tonotopic axis, is influenced by afferent activity, and facilitates glutamate clearance. J Comp Neurol. 2009; 514: 92–106. doi: 10.1002/cne.21998 PMID: 19260071

52. Jalabi W, Kopp-Scheinpflug C, Allen PD, Schiavon E, DiGiacomo RR, Forsythe ID, et al. Sound Localization Ability and Glycinergic Innervation of the Superior Olivary Complex Persist after Genetic Deletion of the Medial Nucleus of the Trapezoid Body. J Neurosci. 2013; 33: 15044–15049. doi: 10.1523/JNEUROSCI.2604-13.2013 PMID: 24048834

53. Altieri SC, Zhao T, Jalabi W, Maricich SM. Development of glycinergic innervation to the murine LSO and SPN in the presence and absence of the MNTB. Front Neural Circuits. 2014; 8: 109. doi: 10.3389/fncir.2014.00109 PMID: 25309335

54. Glendenning KK, Masterton RB, Baker BN, Wenthold RJ. Acoustic chiasm III: Nature, distribution, and sources of afferents to the lateral superior olive in the cat. J Comp Neurol. 1991; 310: 377–400. doi: 10.1002/cne.903100308 PMID: 1723989

55. Kuwabara N, Zook JM. Projections to the medial superior olive from the medial and lateral nuclei of the trapezoid body in rodents and bats. J Comp Neurol. 1992; 324: 522–538. doi: 10.1002/cne.903240406 PMID: 1430335

56. Joris PX, Yin TC. Envelope coding in the lateral superior olive. I. Sensitivity to interaural time differences. J Neurophysiol. 1995; 73: 1043–1062. PMID: 7608754

57. Park TJ, Grothe B. From pattern recognition to sound localization: A by-product of growing, larger during evolution. Naturwissenschaften. 1996; 83: 30–32. doi: 10.1007/BF01139307 PMID: 8637604

58. Grothe B, Park TJ, Schuller G. Medial Superior Olive in the Free-Tailed Bat: Response to Pure Tones and Amplitude-Modulated Tones. J Neurophysiol. 1997; 77: 1553–1565. PMID: 9084619

59. Kadner A, Berrebi AS. Encoding of temporal features of auditory stimuli in the medial nucleus of the trapezoid body and superior paraolivary nucleus of the rat. Neuroscience. 2008; 151: 868–887. doi: 10.1016/j.neuroscience.2007.11.008 PMID: 18155850

60. Felix RA, Fridberger A, Leijon S, Berrebi AS, Magnusson AK. Sound Rhythms Are Encoded by Postinhibitory Rebound Spiking in the Superior Paraolivary Nucleus. J Neurosci. 2011; 31: 12566–12578. doi: 10.1523/JNEUROSCI.2450-11.2011 PMID: 21880918

61. Wahl-Schott C, Biel M. HCN channels: Structure, cellular regulation and physiological function. Cell Mol Life Sci. 2009; 66: 470–494. doi: 10.1007/s00018-008-8525-0 PMID: 18953682

62. Yamada R, Kuba H, Ishii TM, Ohmori H. Hyperpolarization-Activated Cyclic Nucleotide-Gated Cation Channels Regulate Auditory Coincidence Detection in Nucleus Laminaris of the Chick. J Neurosci. 2005; 25: 8867–8877. doi: 10.1523/JNEUROSCI.2541-05.2005 PMID: 16192376

63. Mathews PJ, Jercog PE, Rinzel J, Scott LL, Golding NL. Control of submillisecond synaptic timing in binaural coincidence detectors by Kv1 channels. Nat Neurosci. 2010; 13: 601–609. doi: 10.1038/nn.2530 PMID: 20364143

64. Golding NL, Oertel D. Synaptic integration in dendrites: Exceptional need for speed. J Physiol. 2012; doi: 10.1113/jphysiol.2012.229328

65. Khurana S, Liu Z, Lewis AS, Rosa K, Chetkovich D, Golding NL. An Essential Role for Modulation of Hyperpolarization-Activated Current in the Development of Binaural Temporal Precision. J Neurosci. 2012; 32: 2814–2823. doi: 10.1523/JNEUROSCI.3882-11.2012 PMID: 22357864

66. Leão KE, Leão RN, Walmsley B. Modulation of dendritic synaptic processing in the lateral superior olive by hyperpolarization-activated currents. Eur J Neurosci. 2011; 33: 1462–1470. doi: 10.1111/j.1460-9568.2011.07627.x PMID: 21366727

67. Benson CG, Cant NB. The ventral nucleus of the lateral lemniscus of the gerbil (Meriones unguiculatus): Organization of connections with the cochlear nucleus and the inferior colliculus. J Comp Neurol. 2008; 510: 673–690. doi: 10.1002/cne.21820 PMID: 18709666

68. Oertel D. The Role of Timing in the Brain Stem Auditory Nuclei of Vertebrates. Annu Rev Physiol. 1999; 61: 497–519. doi: 10.1146/annurev.physiol.61.1.497 PMID: 10099699

69. Recio-Spinoso A, Joris PX. Temporal properties of responses to sound in the ventral nucleus of the lateral lemniscus. J Neurophysiol. 2014; 111: 817–835. doi: 10.1152/jn.00971.2011 PMID: 24285864

70. Trattner B, Gravot CM, Grothe B, Kunz L. Metabolic Maturation of Auditory Neurones in the Superior Olivary Complex. PLoS ONE. 2013; 8: e67351. doi: 10.1371/journal.pone.0067351 PMID: 23826275

71. Lange S, Burda H, Wegner RE, Dammann P, Begall S, Kawalika M. Living in a "stethoscope": burrow-acoustics promote auditory specializations in subterranean rodents. Naturwissenschaften. 2007; 94: 134–138. doi: 10.1007/s00114-006-0168-0 PMID: 17119910

72. Jarvis JU. Eusociality in a mammal: cooperative breeding in naked mole-rat colonies. Science. 1981; 212: 571–573. PMID: 7209555

73. Yosida S, Kobayasi KI, Ikebuchi M, Ozaki R, Okanoya K. Antiphonal Vocalization of a Subterranean Rodent, the Naked Mole-Rat (Heterocephalus glaber). Ethology. 2007; 113: 703–710. doi: 10.1111/j.1439-0310.2007.01371.x
